# Associations between suicide attempts and clinical, metabolic, and inflammatory markers in Chinese patients with long-term schizophrenia

**DOI:** 10.3389/fpsyt.2025.1651246

**Published:** 2025-09-16

**Authors:** Lili Zhao, Lewei Liu, Xu Zhang, Xi Zhang, Xianhu Yao, Wenzheng Li, Lei Xia, Huanzhong Liu

**Affiliations:** ^1^ Department of Psychiatry, The Fourth Affiliated Hospital of Anhui Medical University, Hefei, Anhui, China; ^2^ Department of Psychiatry, School of Mental Health and Psychological Sciences, Anhui Medical University, Hefei, Anhui, China; ^3^ Department of Psychiatry, Ma’anshan Fourth People’s Hospital, Ma’anshan, Anhui, China; ^4^ Department of Psychiatry, Hefei Fourth People’s Hospital, Hefei, Anhui, China

**Keywords:** long-term schizophrenia, suicide attempt, inflammatory cytokines, metabolism, clinical symptoms

## Abstract

**Background:**

Suicide attempt (SA) is common among patients with long-term schizophrenia (SCZ), but the mechanisms underlying its occurrence remain incompletely understood. Thus, the purpose of this study was to investigate the associations between SA and clinical, metabolic, and inflammatory markers in Chinese patients with SCZ.

**Methods:**

This study enrolled 299 patients with SCZ. SA, psychotic symptoms, depressive symptoms, and insomnia were evaluated through standardized questions, the Positive and Negative Syndrome Scale (PANSS), the Calgary Depression Scale for Schizophrenia (CDSS), and the Insomnia Severity Index (ISI), respectively. In addition, we measured metabolic parameters including total cholesterol (TC), triglycerides (TG), high-density lipoproteins (HDL) and low-density lipoproteins (LDL), fasting blood glucose (FBG), and fasting insulin (FI), along with inflammatory cytokine levels, including interleukin (IL)-1β, IL-6, IL-17A and tumor necrosis factor-α (TNF-α). Univariate analyses (chi-square test, the independent samples t-test or the Mann-Whitney U-test) were followed by multivariable logistic regression (“Forward: LR”) to identify independent risk factors for SA. Log10-transformed values were applied to inflammatory-cytokine data to approximate normal distribution. All analyses were performed in SPSS 23.0; *P* < 0.05 (two-sided) was considered statistically significant.

**Results:**

The prevalence of SA in patients with SCZ was 22.7%. Patients in the SA group had a higher proportion of females, more severe depressive symptoms, and higher levels of TC, TG, Log IL-1β, and Log IL-6 (all *P* < 0.05). Logistic regression analyses showed that gender (OR = 0.239, 95% CI = 0.127 - 0.450, *P* < 0.001), CDSS total score (OR = 1.250, 95% CI = 1.146 - 1.364, *P* < 0.001), TC (OR = 1.682, 95% CI = 1.178 - 2.402, P = 0.004), and Log IL-1β (OR = 2.225, 95% CI = 1.114 - 4.564, *P* = 0.024) were independent correlates of SA.

**Conclusions:**

Female gender, greater depressive severity, and elevated metabolic and proinflammatory markers (specifically TC and IL-1β) were independently associated with increased risk of SA in Chinese patients with long-term SCZ. These findings suggest that future interventions targeting metabolic and inflammatory pathways may hold promise for preventing SA in this population.

## 1 Introduction

Schizophrenia (SCZ) is a chronic mental illness that involves impaired cognitive functioning and behavioral patterns (1). Among patients with SCZ, suicide constitutes the predominant cause of death (2). When compared to the general population, the suicide rate among patients with SCZ is nearly 10 times higher (3). Suicidal behavior typically progresses through multiple stages, among which suicide attempt (SA) constitute a critical stage and a robust predictor of subsequent suicide (4). SA is defined as deliberate self-harm with non-fatal outcome and apparent or inferred intent to die (5). Individuals at this stage may already be in a state of extreme distress or despair and may attempt suicide again (6, 7). Thus, identifying the risk factors associated with SA is of critical for reducing the suicide rate among patients with SCZ and improving their prognosis.

First, SA may be associated with sociodemographic and clinical symptoms. For instance, a comprehensive meta-analysis of 96 studies identified smoking history, alcohol use, depressive symptoms, and a family history of suicide as significant risk factors for SA in patients with SCZ (8). Similarly, evidence from a Chinese cross-sectional investigation also demonstrated that SA was closely related to age, smoking, and depressive symptoms (9). Another meta-analysis further revealed that insomnia in patients with SCZ was strongly associated with a significantly increased risk of SA (10). However, most of the studies on SA in patients with SCZ are from Western countries. Due to differences in sociocultural, economic backgrounds and healthcare systems, the results of studies in Western countries may not be directly generalizable to Chinese patients (11). Currently, there is relatively few research on SA among patients with SCZ in China, which need to be further explored with more research.

It is worth noting that SA in patients with SCZ may also be related to certain abnormal changes in biological markers. In recent years, several studies have focused on the associations between metabolism and suicidal behaviors (12, 13). For example, a systematic evaluation and meta-analysis reported that SA in patients with SCZ was associated with lower total cholesterol (TC) levels (14). Another study on patients with depressive disorders suggested that decreased low-density lipoprotein (LDL) levels may be related to SA (15). Conversely, a cross-sectional investigation noted that there is currently no evidence supporting a correlation between recent SA and lipid lparameters, including TC, triglycerides (TG), and LDL, in patients with mental disorders (16). Given these inconsistent findings in this field and the limited research exploring the associations between SA and metabolic markers in patients with SCZ, further investigation is warranted.

Inflammatory cytokines are also contribute significantly to the etiopathogenesis of SCZ. Existing research indicates that a chronic inflammatory state exists in patients with long-term SCZ, which is manifested by elevated levels of inflammatory cytokines (17, 18). At the same time, some studies have found significantly elevated levels of certain inflammatory cytokines in suicide attempters and completers. For example, a cross-sectional study found that both recent and distant suicidal behaviors in patients with depressive disorders were associated with elevated interleukin (IL)-6 levels (19). Janelidze et al. reported significantly increased blood levels of IL-6 and tumor necrosis factor-alpha (TNF-α) among individuals with SA (20). Furthermore, a meta-analysis of individuals with psychiatric disorders revealed that patients with mental disorders and immune activation have a higher risk of SA, which may be related to increased neurotoxicity caused by inflammation and oxidative stress (21). These studies support the potential role of inflammatory cytokines in suicidal behaviors. However, most of the above studies have focused on patients with depressive disorders. Although the role of inflammatory cytokines in SCZ has been explored, research on the associations between these factors and SA remains relatively scarce and has not been fully elucidated.

Therefore, this study aimed to explore the associations between SA and clinical, metabolic and inflammatory markers in patients with long-term SCZ, with a view to providing new perspectives on understanding the complex mechanisms of suicidal behavior in long-term SCZ while providing new ideas for clinical intervention.

## 2 Methods

### 2.1 Study design and participants

This cross-sectional study focused on individuals with long-term SCZ who were admitted to The Fourth Affiliated Hospital of Anhui Medical University, Hefei Fourth People’s Hospital, and Ma’anshan Fourth People’s Hospital between May and December 2018. The inclusion criteria of the study subjects were: (1) between the ages of 18 and 65; (2) diagnosis of SCZ was independently confirmed by at least two attending-level physicians using the Diagnostic and Statistical Manual of Mental Disorders, fifth edition (DSM-5) criteria, and illness duration exceeded five years; and (3) possessed the appropriate comprehension ability to complete the study assessment. Exclusion criteria were: (1) current or previous diagnosis of other psychiatric disorders such as depressive disorders, bipolar disorder, etc.; (2) existence of concurrent infections, severe neuroendocrine or metabolic conditions, and other significant health issues; and (3) taking non-steroidal anti-inflammatory drugs, glucocorticosteroids, or other immune-modulating agents. The study initially enrolled 334 patients with long-term SCZ who met the inclusion criteria; 299 of them completed all study assessments (35 had missing key information).

The study received approval from the ethics board at The Fourth Affiliated Hospital of Anhui Medical University (No. 201805-kyxm-03), and all participants and their guardians provided written consent.

### 2.2 Measures

#### 2.2.1 Demographic characteristics

Demographic and clinical data were collected via a self-report questionnaire covering gender, age, body mass index (BMI), systolic and diastolic blood pressure, age at onset, duration of illness, and antipsychotic use. Antipsychotic dosages were converted to chlorpromazine equivalents (22).

#### 2.2.2 Suicide attempt

This study assessed patients’ SA by adopting a standardized question, “Have you ever attempted suicide?” Those who answered “yes” were classified as the SA group (23). Family members were also interviewed to corroborate responses and reduce under-reporting (24).

#### 2.2.3 Psychotic symptom

The Positive and Negative Symptom Scale (PANSS) was used to assess psychotic symptoms (25). The scale comprises 30 items, each rated from 1 to 7. The five-factor model of PANSS, including positive, negative, cognitive, arousal, and depression, was used in this study (26). Currently, the PANSS has good reliability and validity in Chinese patients with SCZ (27).

#### 2.2.4 Depressive symptom

The Calgary Depression Scale (CDSS) was used to evaluate the severity of depressive symptoms (28). The scale comprises nine items, with total scores ranging from 0 to 27. Higher total scores indicate more severe depressive symptoms. It has demonstrated excellent reliability and validity and has been widely applied in Chinese patients with SCZ (29).

#### 2.2.5 Insomnia

The Insomnia severity index (ISI) was used to assess insomnia (30). Comprising seven items, each rated 0 - 4, the scale yields higher scores that indicate greater insomnia severity. The Chinese version of the ISI has good reliability, validity and internal consistency (31).

#### 2.2.6 Biochemical assays

Fasting blood samples were collected between 7:00 and 8:00 a.m. after an overnight fast. Within 30 min of collection, samples were centrifuged at 3000 rpm for 15 minutes at 4 °C. The plasma was separated and stored in sterile tubes at -80 °C before detection. TC, TG, high-density lipoproteins (HDL), LDL, fasting blood glucose (FBG) and fasting insulin (FI) levels were tested using Siemens Advia Chemistry XPT automatic biochemistry analyzer (Siemens, New York, USA). Plasma levels of inflammatory cytokines IL-1β, IL-6, IL-17A, and TNF-α were detected by enzyme-linked immunosorbent assay (ELISA) (kit: Sangong Biotech, Shanghai, China).

### 2.3 Statistical analyses

SPSS 23.0 was used for statistical analyses. Categorical variables were expressed as frequencies (%). The Kolmogorov-Smirnov test was used to test whether continuous variables conformed to normal distribution. Conformity to normal distribution was expressed as mean ± standard deviation (SD), and non-normal distribution was expressed as median (quartiles) [M (Q1, Q3)]. In univariate analyses, the chi-square test, the independent samples t-test, and the Mann-Whitney U-test were used to compare demographic and clinical variables between the SA group and the no SA group. Subsequently, logistic regression models using the “ Forward: LR” approach were used to determine independent risk factors for SA. In these analyses, to ensure that the measurements of inflammatory cytokines conformed to a normal distribution, we referred to previous studies (32, 33) and log-transformed the levels of IL-1β, IL-6, IL-17A, and TNF-α on a base of 10 to obtain Log (X) values for Log IL-1β, Log IL-6, Log IL-17A, and Log TNF-α. For all statistical tests, *P* < 0.05 (two-sided) was defined as statistically significant.

## 3 Results

### 3.1 Comparison of sociodemographic and clinical data between patients in the SA and no SA groups in long-term SCZ

**Figure 1:**
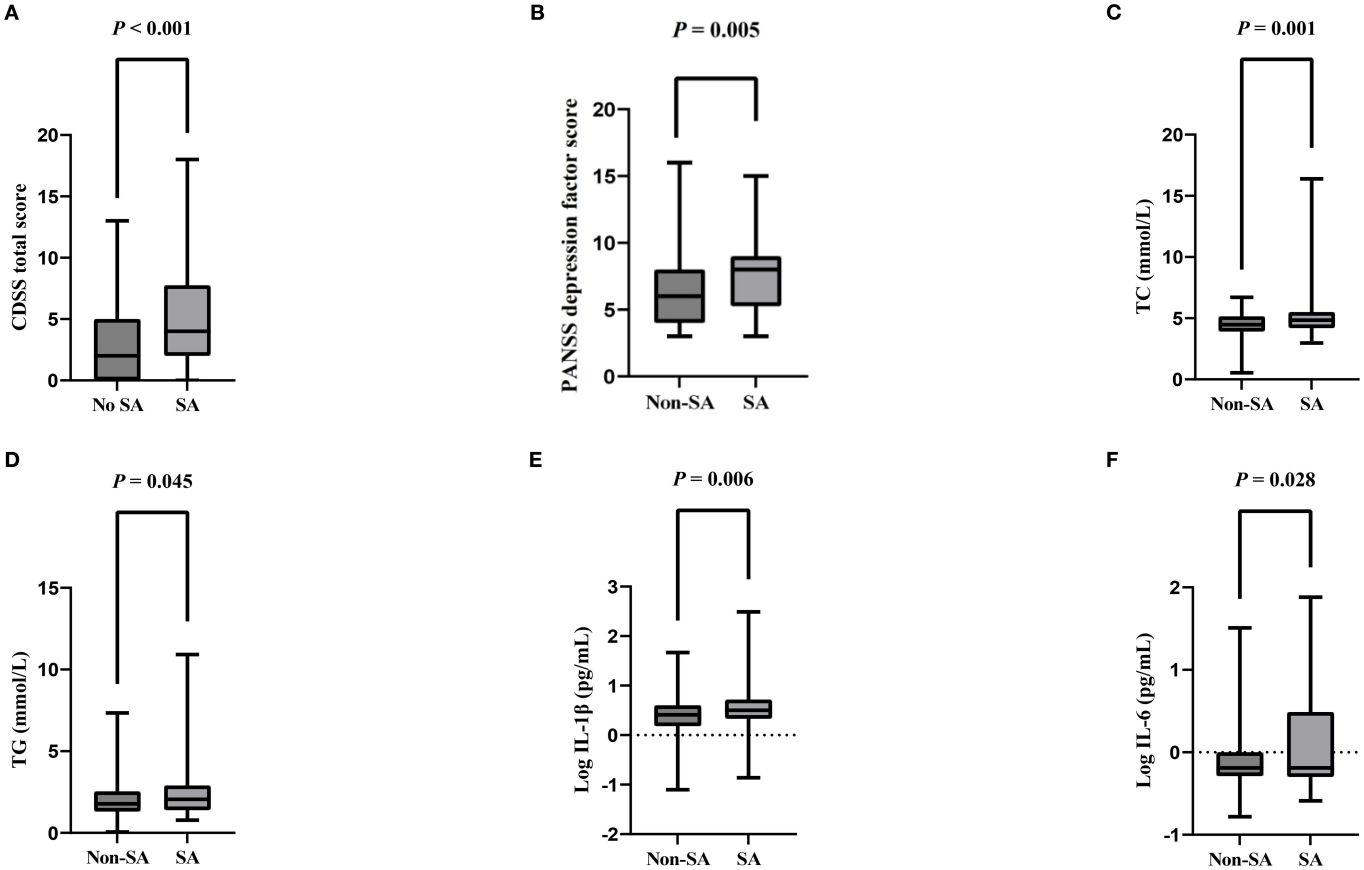
Comparison of CDSS total score, PANSS depression factor score, TC, TG, Log IL-1β, Log IL-6 levels in the SA and no SA groups. [**(A)** Comparison of CDSS total score in the SA and no SA groups; **(B)** Comparison of PANSS depression factor score in the SA and no SA groups; **(C)** Comparison of TC in the SA and no SA groups; **(D)** Comparison of TG in the SA and no SA groups; **(E)** Comparison of Log IL-1β in the SA and no SA groups; **(F)** Comparison of Log IL-6 levels in the SA and no SA groups]. SA, Suicide Attempt; CDSS, Calgary Depression Scale for Schizophrenia; PANSS, The Positive and Negative Syndrome Scale; TC, Total Cholesterol; TG, Triglycerides; IL-1β, Interleukin-1β; IL-6, Interleukin-6.

In this study, the prevalence of SA was 22.7% (68/299). Univariate analyses showed a higher proportion of females in the SA group (χ² = 22.119, *P* < 0.001) and higher CDSS total score (Z = -4.103, *P* < 0.001) and PANSS depression factor scores (t = -2.798, *P* = 0.005), compared to the group without SA. In terms of biochemical markers, the levels of TC (t = -3.483, *P* = 0.001), TG (t = -2.010, *P* = 0.045), Log IL-1β (t = -2.752, *P* = 0.006) and Log IL-6 (t = -2.238, *P* = 0.028) were higher in the SA group (**Table 1** and **Figure 1**).

**Table 1:** Comparison of sociodemographic and clinical data between patients in the SA and no SA groups in long-term SCZ.

Variables	Total (N=299)	No SA (n =231)	SA (n = 68)	t/Z/ χ^2^	*P*
Females, n (%)	120 (40.13)	76 (32.90)	44 (64.71)	22.119^a^	**< 0.001**
Age (years), mean (SD)	44.86 (11.61)	45.05 (12.04)	44.19 (10.09)	0.537	0.592
BMI (kg/m^2^), mean(SD)	24.03 (3.83)	24.01 (3.88)	24.10 (3.67)	-0.161	0.872
Systolic blood pressure (mmHg), mean(SD)	114.89 (12.43)	114.75 (12.55)	115.34 (12.11)	-0.341	0.734
Diastolic blood pressure (mmHg), mean(SD)	76.12 (8.12)	76.50 (8.51)	74.81 (6.48)	1.515	0.131
Age at onset (years), mean(SD)	26.05 (8.46)	26.18 (8.62)	25.60 (7.96)	0.491	0.623
Duration of illness (years), mean(SD)	18.81 (10.29)	18.92 (10.55)	18.44 (9.43)	0.338	0.736
Chlorpromazine Equivalents (mg), mean(SD)	453.10 (251.09)	448.02 (242.97)	470.33 (278.15)	-0.643	0.521
ISI total score, median (Q1, Q3)	2.00 (1.00, 5.00)	2.00 (1.00, 5.00)	2.00 (1.00, 6.75)	-1.661^b^	0.097
CDSS total score, median (Q1, Q3)	3.00 (1.00, 5.00)	2.00 (0.00, 5.00)	4.00 (2.00, 7.75)	-4.103^b^	**< 0.001**
PANSS total score, mean(SD)	78.03 (24.33)	78.42 (24.68)	76.71 (23.22)	0.511	0.609
Positive score, mean(SD)	10.68 (5.05)	10.46 (5.03)	11.43 (5.06)	-1.385	0.167
Negative score, mean(SD)	17.64 (7.05)	17.97 (7.19)	16.51 (6.51)	1.494	0.136
Cognitive/Disorganized score, mean(SD)	9.00 (2.93)	9.12 (3.04)	8.60 (2.51)	1.411	0.161
Excited score, mean(SD)	7.93 (3.68)	7.89 (3.82)	8.07 (3.19)	-0.394	0.694
Depressive score, mean(SD)	6.86 (2.81)	6.62 (2.72)	7.69 (2.97)	-2.798	**0.005**
Metabolic markers					
TC (mmol/L), mean(SD)	4.62 (1.15)	4.50 (0.92)	5.04 (1.66)	-3.483	**0.001**
TG (mmol/L), mean(SD)	2.17 (1.31)	2.09 (1.20)	2.45 (1.62)	-2.010	**0.045**
HDL (mmol/L), mean(SD)	1.05 (0.26)	1.04 (0.26)	1.09 (0.28)	-1.400	0.163
LDL (mmol/L), mean(SD)	2.37 (0.58)	2.35 (0.59)	2.45 (0.55)	-1.342	0.180
FBG (mmol/L), mean(SD)	5.04 (1.31)	5.03 (1.36)	5.07 (1.12)	-0.218	0.828
FI (µIU/mL), mean(SD)	9.46 (7.93)	9.46 (8.32)	9.43 (6.52)	0.032	0.974
Inflammatory cytokines					
Log IL-1β (pg/mL), mean(SD)	0.43 (0.42)	0.40 (0.39)	0.55 (0.49)	-2.752	**0.006**
Log IL-6 (pg/mL), mean(SD)	-0.01 (0.49)	-0.05 (0.44)	0.12 (0.59)	-2.238	**0.028**
Log IL-17A (pg/mL), mean(SD)	0.70 (0.57)	0.67 (0.54)	0.80 (0.67)	-1.398	0.165
Log TNF-α (pg/mL), mean(SD)	1.29 (0.21)	1.28 (0.18)	1.32 (0.27)	-1.191	0.237

SA, Suicide Attempt; SCZ, schizophrenia; BMI, Body Mass Index; ISI, Insomnia Severity Index; CDSS, Calgary Depression Scale for Schizophrenia; PANSS, The Positive and Negative Syndrome Scale; TC, Total Cholesterol; TG, Triglycerides; HDL, High-Density Lipoprotein; LDL, Low-Density Lipoprotein; FBG, Fasting Blood Glucose; FI, Fasting Insulin; IL-1β, Interleukin-1β; IL-6, Interleukin-6; IL-17A, Interleukin-17A; TNF-α, Tumour Necrosis Factor-α; SD, Standard Deviation; median (Q1, Q3), Median with Interquartile Range; t, Independent Samples t-test; Z, Mann-Whitney U-test statistic; χ^2^, Chi-Square Test;

^a^Chi-Square Test; ^b^Mann-Whitney U-test;

Bold values mean *P* < 0.05.

### 3.2 Independent factors associated with SA by multivariate logistic stepwise regression analyses

**Table 2** displayed the findings from the multivariate logistic stepwise regression analyses. Gender (OR = 0.239, 95% CI = 0.127 - 0.450, *P* < 0.001), CDSS total score (OR = 1.250, 95% CI = 1.146 - 1.364, *P* < 0.001), TC (OR = 1.682, 95% CI = 1.178 - 2.402, *P* = 0.004), and Log IL-1β (OR = 2.225, 95% CI = 1.114 - 4.564, *P* = 0.024) were independent correlates of SA in patients with long-term SCZ.

**Table 2:** Independent factors associated with SA by multivariate logistic stepwise regression analyses.

Variables	B	SE	Wald χ2	OR	95% CI	*P*
Gender	-1.431	0.323	19.646	0.239	0.127 - 0.450	**< 0.001**
CDSS total score	0.223	0.044	25.266	1.250	1.146 - 1.364	**< 0.001**
TC	0.520	0.182	8.179	1.682	1.178 - 2.402	**0.004**
Log IL-1β	0.813	0.360	5.107	2.225	1.114 - 4.564	**0.024**

SA, Suicide Attempt; CDSS, Calgary Depression Scale for Schizophrenia; TC, Total Cholesterol; IL-1β, Interleukin-1β; SE, Standard Error; OR, Odds Ratio; CI, Confidence Interval;

Bold values mean *P* < 0.05.

## 4 Discussion

The current investigation explored risk factors associated with SA in patients with long-term SCZ. Initial analyses revealed a 22.7% prevalence of SA among the patients. This rate is higher than the lifetime prevalence of 14.6% reported among Chinese patients with SCZ in a prior meta-analysis (34). This discrepancy may be attributable to our study’s focus on hospitalized patients, who typically experience more severe illness and thus may be at greater risk of SA. Further analyses revealed that gender was strongly associated with SA, with female patients having a higher rate of SA. This finding was consistent with the results of a cross-sectional study (35). Similarly, a prospective cohort study also pointed out that female gender is a significant predictor of SA (36). Several mechanisms may explain this association. First, neuroendocrine factors, such as dramatic fluctuations in female hormone levels (e.g., during the perimenstrual period), may exacerbate mood swings and increase susceptibility to depression and anxiety, thereby elevating suicide risk (37). Second, women may be more inclined to internalize emotions, leading to an accumulation of psychological distress and an increased vulnerability to suicide (38). Finally, sociocultural factors should not be ignored, as females may face greater social pressures and role conflicts, and these factors work together to lead to an increased risk of suicide in female patients. Therefore, it is crucial in clinical practice to closely monitor the suicide risk among female patients with SCZ and to implement timely, targeted interventions to reduce the incidence of SA.

Regarding clinical symptoms, depressive symptoms were identified as a significant predictor of SA among individuals with long-term SCZ, a finding consistent with numerous studies (9, 39). Yamada et al. discovered that 36.4% of patients developed depressive symptoms two years after the onset of SCZ, which also predicted a worse future clinical outcome for patients with SCZ (40). Compared to patients with less than five years of illness, those with six to ten years of illness were two times more likely to have concurrent depression (41). This implies that the longer the duration of SCZ, the higher the likelihood of developing comorbid depression, and since suicide is one of the clinical symptoms of depression, the presence of depression may also exacerbate suicidal intent in patients with SCZ. A cross-sectional study from Egypt revealed that the severity of depressive symptoms was significantly linked to a higher risk of suicide in individuals with SCZ (42). This study further confirmed this view, indicating that depressive symptoms may be an important early warning sign of suicidal behaviors in patients with SCZ. Notably, depressive symptoms are often misinterpreted as the negative symptoms of SCZ, leading to underdiagnosis and inadequate treatment (43). Consequently, in clinical practice, patients with SCZ should be regularly screened for depressive symptoms, and depressed mood should be promptly recognized to allow for the implementation of targeted comprehensive interventions to mitigate this risk. Specifically, appropriate evidence-based psychotherapy may be selected alongside medication treatment. For example, Cognitive Behavioral Therapy (CBT) and Interpersonal Therapy (IPT) have been shown to improve individuals’ depressive symptoms (44, 45). This integrated intervention combining medication with psychotherapy, enables more effective identification, assessment, and management of depressive symptoms, thereby reducing suicide risk.

Unfortunately, this study did not identify a significant association between SA and insomnia among individuals suffering from long-term SCZ. This finding contrasted with the results of previous studies. For instance, one large-scale study found insomnia correlated with a notable 2.7 times increase in the odds of current suicidal ideation, and a 5.5 times increase in the odds of SA in the past 6 months (46). A cross-sectional study that included patients with SCZ over the age of 50 also demonstrated that elderly patients with SA had significantly higher insomnia symptoms (47). Prolonged sleep deprivation associated with insomnia may amplify the impact of psychiatric symptoms on patients, exacerbating their psychological distress and cognitive dysfunction, thereby increasing the likelihood of SA (48). These inconsistent findings may be attributed to different research methods, sample heterogeneity, and the confounding effects of medication. This was a cross-sectional study, and the sample mainly focused on the group of long-term schizophrenic, who generally had a long course of illness and whose disease had entered a relatively stable stage. Their SA may be more strongly influenced by long-term accumulated disease factors, social functional impairment, and sustained pharmacotherapy, with the role of insomnia being attenuated or obscured by these more dominant variables. For instance, a systematic review showed that second-generation antipsychotics such as clozapine and olanzapine improved insomnia in patients with SCZ (49). Consequently, further investigation is required to clarify the complex relationship between insomnia and SA in SCZ.

In addition, this study observed that patients who attempted suicide had higher levels of TC and TG. Regression analysis identified TC as an independent risk factor for SA, suggesting that metabolic dysregulation may contribute to suicidal behavior. Similarly, a cross-sectional study noted that patients with mental disorders who had lower levels of TC also had lower rates of SA (16). Another study from China showed that higher TC levels are a risk factor for suicide in schizophrenics (12). This may be due to the fact that cholesterol is an important component of nerve cell membranes, and abnormal levels may affect the synthesis and function of neurotransmitters (e.g., serotonin, dopamine, etc.), which in turn may affect mood regulation and impulse control, increasing the risk of suicide (50, 51). Nevertheless, there were also studies that have reached different conclusions from ours or even presented opposite views. For example, some studies have shown that no significant associations between TC levels and SA in patients with SCZ (16, 52). While another cross-sectional study pointed out that, compared with patients who did not attempt suicide, TC levels were significantly lower in those who attempted suicide (53). Given these contradictory findings, further research is essential to elucidate the precise role of lipid metabolism in suicide risk among individuals with SCZ.

Finally, we also observed the elevated levels of inflammatory cytokines (including IL-1β and IL-6) in the SA group, with IL-1β emerging as a significant independent predictor of SA. A meta-analysis revealed considerably higher concentrations of IL-1β and IL-6 in blood and postmortem brain samples of individuals with mental disorders who had attempted suicide (54). This discovery aligns with the outcomes of our study and further supports the potential influence of inflammatory cytokines on suicidal behavior in individuals with long-term SCZ. Chronic inflammatory states may affect brain function through multiple pathways. As outlined in the review by Miller & Raison, proinflammatory cytokines can profoundly disrupt the neurotransmitter systems (e.g., serotonin and glutamate metabolism) and neurocircuitry (e.g., involving the basal ganglia and prefrontal cortex) that are critically involved in mood regulation and impulsivity (55). Specifically, inflammatory cytokines may lead to elevated levels of neurotoxic metabolites (e.g., quinolinic acid) through activation of the kynurenine pathway, which in turn affects neuroinflammation and glutamate metabolism, leading to mood and behavioral abnormalities and a heightened risk of suicide (56). Concurrently, inflammatory cytokines can disrupt serotonin synthesis and release, leading to decreased serotonin levels, increased individual impulsivity, and thus increased suicide risk (57). We hypothesize that this inflammatory state may both exacerbate depressive symptoms and underlie observed metabolic dysregulation, creating a pathogenic cycle that increased the risk of suicide. Collectively, these findings suggest that the assessment of suicide risk in patients with long-term SCZ can also be approached from an inflammatory perspective, providing new ideas for interventions for suicide prevention in this patient population, such as modulating the inflammatory state to reduce suicide risk.

The present study explored the factors associated with SA in patients with long-term SCZ from both clinical symptoms and biological markers perspectives, providing new insights into understanding the mechanisms by which suicidal behavior occurs in this patient population. However, this study had certain constraints: (1) The design of this study was cross-sectional and did not allow for causal inferences, and more longitudinal studies are needed in the future to investigate the contributing factors for SA in patients with long-term SCZ. (2) For the assessment of SA, we relied mainly on clinical observations and patients’ self-reports, which may be subject to concealment and subjective bias. (3) This study was conducted in only three hospitals in Anhui Province, which may limit the generalization of the findings.

## 5 Conclusion

In summary, this study demonstrated a high prevalence of SA among patients with long-term SCZ, with female gender, greater severity of depressive symptoms, and elevated levels of metabolic and proinflammatory markers (specifically TC and IL-1β) identified as significant independent risk factors. These findings have direct clinical implications. Firstly, they underscore the critical need for systematic and regular screening for depressive symptoms in this population, with particular attention to female patients. Secondly, routine monitoring of metabolic parameters (such as TC) and proinflammatory biomarkers (like IL-1β) should be integrated into the clinical management of patients with long-term SCZ to aid in suicide risk assessment. In practice, the assessment of suicidal risk should not be limited to self-reported suicidal ideation but should be combined with these key clinical features and biomarkers. Finally, early intervention strategies targeting depressive symptoms and these modifiable biological risk factors (e.g., through pharmacological or lifestyle interventions) could be pivotal in suicide prevention. Moving forward, a more accurate suicide risk prediction model can be established by combining clinical symptoms, biochemical markers, and other multidimensional information. This will ensure that patients at the greatest risk can be identified early and receive prompt, personalized interventions.

## Data Availability

The raw data supporting the conclusions of this article will be made available by the authors, without undue reservation.
